# Exploring Knee Alignment: Demystifying Traditional and Emerging Approaches

**DOI:** 10.7759/cureus.91423

**Published:** 2025-09-01

**Authors:** Mohammed Elmajee, Mahmoud Mersal, Walid Ben Nafa, Ahmed Elsayed, Mohamed Alsonbaty, Islam A Sherif, Mohamed Mahmoud

**Affiliations:** 1 Trauma and Orthopaedics, Royal Orthopaedic Hospital NHS Foundation Trust, Birmingham, GBR; 2 Trauma and Orthopaedics, University Hospitals Birmingham NHS Foundation Trust, Birmingham, GBR; 3 Trauma and Orthopaedics, St Helens Hospital, St Helens, GBR; 4 Trauma and Orthopaedics, Manchester Royal Infirmary, Manchester, GBR; 5 Trauma and Orthopaedics, Queen Elizabeth Hospital Birmingham, Birmingham, GBR; 6 Trauma and Orthopaedics, Warwick Hospital, Warwick, GBR

**Keywords:** adjusted mechanical alignment, functional alignment, kinematic alignment, knee replacement surgery, mechanical alignment, patient satisfaction tka, personalized knee alignment, restricted kinematic alignment (rka), soft tissue balancing, total knee arthroplasty

## Abstract

Knee alignment plays a pivotal role in the outcomes of total knee replacement (TKR), influencing postoperative function, pain reduction, and long-term implant longevity. Over the past few decades, various knee alignment philosophies have been proposed to optimize surgical results, including the mechanical axis and kinematic alignment (KA). This review provides a comprehensive analysis of these alignment philosophies, evaluating their theoretical foundations, clinical outcomes, and impact on TKR outcomes, with a particular focus on the emerging role of KA.

The mechanical axis, traditionally regarded as the standard for TKR, ensures balance and stability by aligning the knee components along the mechanical axis of the lower limb. However, recent studies have questioned the universality of this approach, particularly considering the coronal plane alignment of the knee (CPAK), CPAK classification, and the growing popularity of the individualized knee arthroplasty concept. In contrast, KA seeks to restore the patient's prearthritic knee alignment, positioning the femoral and tibial components in accordance with the natural motion and geometry of the knee. Emerging evidence supports KA as a promising technique, demonstrating increased patient satisfaction and improved functional outcomes compared to traditional alignment methods.

Restricted kinematic alignment (rKA), a more constrained variant of KA, aims to preserve natural joint mechanics while preventing extreme alignments that could lead to instability or accelerated wear. While studies suggest that rKA may offer improved outcomes over the mechanical axis, its applicability and safety remain subjects of ongoing investigation.

This review critically evaluates various knee alignment philosophies, synthesizing contemporary evidence regarding their efficacy in TKR. It places a particular emphasis on the emerging technique of kinematic alignment (KA), highlighting its potential to offer superior outcomes in terms of patient satisfaction, functional recovery, and implant longevity. The findings suggest that, while no single alignment strategy is universally superior, a more individualized, patient-specific approach, particularly one that incorporates kinematic alignment (KA), may lead to enhanced TKR outcomes. This review underscores the need for continued research to refine these alignment strategies and optimize TKR results across a diverse patient population.

## Introduction and background

Knee alignment plays a pivotal role in the outcomes of total knee replacement (TKR), influencing postoperative function, pain reduction, and long-term implant longevity. Over the past few decades, various knee alignment philosophies have been proposed to optimize surgical results, including the mechanical axis and kinematic alignment (KA). This review provides a comprehensive analysis of these alignment philosophies, evaluating their theoretical foundations, clinical outcomes, and impact on TKR outcomes, with a particular focus on the emerging role of KA.

The mechanical axis, traditionally regarded as the standard for TKR, ensures balance and stability by aligning the knee components along the mechanical axis of the lower limb. However, recent studies have questioned the universality of this approach, particularly considering the coronal plane alignment of the knee (CPAK), CPAK classification, and the growing popularity of the individualized knee arthroplasty concept. In contrast, KA seeks to restore the patient's prearthritic knee alignment, positioning the femoral and tibial components in accordance with the natural motion and geometry of the knee. Emerging evidence supports KA as a promising technique, demonstrating increased patient satisfaction and improved functional outcomes compared to traditional alignment methods.

Restricted kinematic alignment (rKA), a more constrained variant of KA, aims to preserve natural joint mechanics while preventing extreme alignments that could lead to instability or accelerated wear. While studies suggest that rKA may offer improved outcomes over the mechanical axis, its applicability and safety remain subjects of ongoing investigation.

Coronal alignment in total knee arthroplasty (TKA) has long been viewed as one of the critical determinants of both implant survival and functional success. For decades, the mechanical alignment (MA) strategy stood as the gold standard, built on the assumption that achieving a neutral limb axis would translate into even load distribution and reduced wear. This principle, born from early implant design and basic engineering logic, became deeply embedded in both training and dogma. However, as implant survivorship improved and surgical volume exploded, it became increasingly clear that mechanical perfection on X-ray did not always translate to patient satisfaction in the clinic.

The disconnect between radiographic alignment and real-world outcomes sparked a reevaluation of what “ideal” alignment should actually mean. KA entered the picture as a disruptive alternative, prioritizing restoration of the patient's native anatomy rather than enforcing a standardized template. By aiming to replicate the prearthritic joint line and preserving soft tissue tension, KA promised a more natural-feeling knee. Yet this came with its own baggage: fears over placing components in extreme positions, concerns about implant longevity, and a steep learning curve for surgeons trained in mechanical dogma.

In response, a spectrum of hybrid approaches began to emerge. Adjusted mechanical alignment (aMA), anatomical alignment (AA), rKA, and, more recently, functional alignment (FA), all attempt to strike a balance, respecting the patient’s native geometry while staying within boundaries thought to reduce risk. What was once a rigid, rule-based system has now evolved into a conversation about personalization, compromise, and context. The rise of computer-assisted technologies and robotics has only accelerated this shift, giving surgeons the tools to plan and execute nuanced strategies with a degree of accuracy previously unimaginable.

This review critically evaluates various knee alignment philosophies, synthesizing contemporary evidence regarding their efficacy in TKR. It places a particular emphasis on the emerging technique of KA, highlighting its potential to offer superior outcomes in terms of patient satisfaction, functional recovery, and implant longevity. The findings suggest that, while no single alignment strategy is universally superior, a more individualized, patient-specific approach, particularly one that incorporates KA, may lead to enhanced TKR outcomes. This review underscores the need for continued research to refine these alignment strategies and optimize TKR results across a diverse patient population.

## Review

Different alignment philosophies in TKR

**Figure 1:**
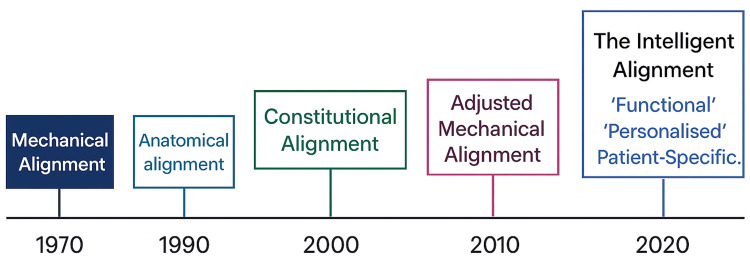
Knee alignment evolution chart Image credit: Created by the authors Mohammed Elmajee and Mahmoud Mersal using ChatGPT-4o image generation (OpenAI, www.openai.com)

The alignment philosophy chosen for TKR plays a crucial role in the long-term survivorship and patient satisfaction of the procedure [1,2]. MA has long been considered the gold standard due to its robust clinical data, but newer approaches such as kinematic and anatomic alignment have gained attention for offering more personalized, natural-feeling knee replacements. While the debate continues over which approach offers the best outcomes, patient-specific factors such as anatomy, activity level, and surgeon expertise should guide the choice of alignment philosophy. Additionally, advances in robotic-assisted surgery and computer navigation are further pushing the boundaries of personalized, FA in TKR [3-6]. Each alignment philosophy has its benefits and limitations, and the choice of technique should be based on the specific needs and anatomy of the patient to achieve the best possible outcomes. These philosophies have evolved over the last half century and will probably continue to do so for the years to come [7,8]. Over the last half-century or so, different philosophies have evolved. Figure 1 illustrates the changing philosophies in alignment through history.

Mechanical alignment

MA is one of the most widely used and traditional approaches in TKR. It aims to position the implant perpendicular to the mechanical axis of the leg, which is a straight line from the center of the hip to the center of the ankle. This method focuses on restoring a neutral, "straight" limb alignment where both the femoral and tibial components are positioned at a 90° angle relative to the mechanical axis. This will ultimately lead to symmetric load distribution, which is thought to enhance implant longevity [4]. MA philosophy aims for fixed alignment targets to create a straight-leg axis [4,5,9]. MA surgeons consider an aligned limb to have an HKA angle within 3° of neutral alignment, whereas a maligned limb falls outside of this safe range [10-12]. For decades, the long-term data support its success in improving implant durability and longevity [13-15]. However, MA and philosophy are increasingly being challenged in recent times and publications due to multiple potential flaws and concerns, particularly with a 15%-20% dissatisfaction rate following MA TKR regardless of the execution methods [16-18].

There are some concerns/issues related to MA TKR, which have been discussed and highlighted in the literature. MA follows a standardized, uniform approach that overlooks individual joint anatomy and natural soft tissue laxity variations. This approach could potentially oversimplify a complex pathology with lots of interplay between 3D bony components and different soft tissue planes and types around the knee. Analyzing 4,884 CT scans of patients with knee pathologies scheduled for total knee arthroplasty (TKA), Almaawi et al. [19] observed that while the average HKA angle was close to neutral (0.1° in varus), there was considerable variation in alignment, ranging from 24° valgus to 25° varus [19]. About 40% of patients showed HKA angles beyond 3°, and 19% exceeded 5°. Only 4% displayed a neutral tibial joint surface (aligned at 0° to the mechanical axis), and just 5% exhibited a neutral distal femoral joint orientation to its mechanical axis [19]. Fewer than 1% of patients had neutral joint surfaces for both the tibia and the femur, which is the objective of MA. To achieve neutral alignment in MA, the distal femur is typically modified by 2.7° on average, with variations ranging from 11° varus to 16° valgus. Similarly, the proximal tibia undergoes a modification averaging 2.9°, spanning from 21° varus to 21 ° valgus [19]. Hirschmann et al. [20] found comparable results in a study of 308 knees without osteoarthritis (OA), noting that the average proximal tibia angle was 2.8° varus, and the distal femur angle was 3.4° valgus [20]. Consequently, the goal of the MA to achieve neutral positioning for both femoral and tibial implants alters the femoral flexion axis, joint line, and entire three-dimensional orientation of the knee joint surfaces for most patients, which could change the joint biomechanics and the clinical outcomes postoperatively [21]. Furthermore, all these variations in the HKA angles measured in healthy individuals or those with osteoarthritic changes were measured in a static mode, and these changes could significantly vary in patients with OA changes, particularly during mobilization, which will ultimately have more impact on the joint biomechanics and ultimately the clinical outcomes [21]. There is emerging evidence in the literature highlighting that some MA TKR knees demonstrated a switch from static varus to dynamic valgus or vice versa during gait, and that utilization of the static HKA angle has no significant correlation with different kinematic forces around the knee [22,23]. The importance of static HKA angle remains uncertain, warranting further investigation.

Furthermore, MA TKR surgeons usually perform an arbitrary posterior tibial slope, simplifying the very complex three-dimensional kinematic movements and interplay between the knee joint at different planes and the soft tissue envelope [24]. Farooq et al. [25] found that restoring the native posterior tibial slope with slight to moderate femoral flexion correlated with more knees “feeling normal,” greater patient satisfaction, and superior patient-reported outcome measure (PROM) scores [25].

The other misconception related to MA TKR is that TKR is a soft-tissue procedure, and ligament and/or soft tissue releases are required to create equal and rectangular gaps both medially and laterally [21]. The orientation of cuts made to the distal femur and proximal tibia creates gaps in the knee's medial and lateral compartments during extension. While there is significant variability in knee anatomy, on average, the femoral joint line sits at around 3° valgus and the tibial joint line at approximately 3° varus [20]. This means that MA often causes substantial anatomical changes for many patients. Uneven removal of bone thickness from each compartment results in extension space imbalances. Blakeney et al. [26] found that the MA TKR approach creates extension space imbalances of 3 mm in 25% of varus knees and 57% of valgus knees, and 5 mm in 8% of varus and 19% of valgus knees [26]. Such imbalances can potentially contribute to joint stiffness, instability, and early loosening of the implant [27]. Clinical outcomes were better when the mediolateral gap difference was 2 mm or less [28]. Therefore, anatomical alterations associated with neutral tibial and femoral cuts under MA can lead to imbalances that, in many cases, are challenging, if not impossible, to resolve through soft tissue releases alone [29]. In a study by Nunley et al. [24], MA-based bone cuts were simulated using a 3D model based on CT scans from 1,000 TKA patients to analyze gap impacts. They observed that, in most cases, whether the knees were varus or valgus, medial and lateral extension gaps were minimized. Specifically, for varus knees, inadequate lateral bone cuts displaced the lateral joint surface downward by an average of 2.1-4.4 mm, while insufficient medial tibial plateau resections raised the medial joint surface by an average of 3.3-1.2 mm for varus and valgus knees, respectively [26]. These alterations result from the MA technique's focus on resecting the most prominent bone surfaces, typically the medial femur and lateral tibia for most patients [26]. Consequently, the imbalance induced by strict MA cuts can only be corrected through ligament releases on the tighter side if MA is to be achieved [30]. Furthermore, raising the joint line could potentially occur with over-resection of the distal femur, such as the case of fixed flexion deformity. In a cadaveric study, another paper found that elevating the medial joint line by 2 and 4 mm led to an increase in coronal mid-flexion laxity by 64% and 111%, respectively [31]. Higher revision rates have been linked to mid-flexion instability [32].

A long-standing belief among many mechanical alignment TKR surgeons is that improving polyethylene wear requires implanting the components perpendicular to the mechanical axis of the lower limb. However, there are several studies that have proved this belief is not necessarily true. For example, one paper examined 501 TKR and compared the 15-year implant survival rates between aligned (HKA = 0° ± 3°) and malaligned (HKA > 3°) groups, finding no statistically significant difference [33]. Another study reported a 7% reoperation rate in KA TKR, which was comparable to, or even lower than, that seen in mechanical alignment TKR [34]. Similar to the American experience, Australian and New Zealand joint registries have shown comparable survivorship and revision rates between KA TKR and MA TKR [35].

In short, MA TKR is still the gold-standard alignment technique applied by lots of TKR surgeons across the globe; however, given all the reasons mentioned above, the authors believe it is time to consider other alternative options and alignment philosophy to allow faster recovery, better functional scores, and pain relief, with similar or better survivorship.

Hybrid alignment (safe-zone principal alignments)

There are techniques and alignment principles where surgeons adopt a middle ground between achieving a standardized approach to all patients regardless of their anatomical and biomechanical differences, MA TKR, and implanting knees as dictated by the individual-knee anatomy and biomechanics. This debate started more than 40 years ago and will probably continue for years to come, with advocates for each alignment claiming that their alignment approach offers the most effective balance of function, longevity, and patient satisfaction in knee replacements. These hybrid alignments include the following.

Anatomical Alignment

**Figure 2:**
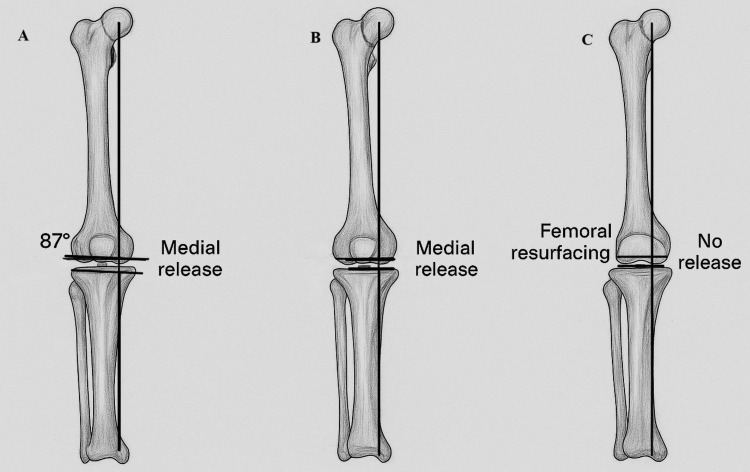
(A) Anatomical alignment, (B) mechanical alignment, and (C) kinematic alignment Image credit: Created by the authors Mohammed Elmajee and Mahmoud Mersal using ChatGPT-4o image generation (OpenAI, www.openai.com) and edited in Adobe Illustrator (Adobe Inc.)

First introduced by Hungerford et al. in the 1980s [36], the technique targets a consistent oblique joint line (2°-3° valgus) relative to the limb's mechanical axis. This is achieved by cutting 3° of varus in the proximal tibial resection and 3° of valgus to the mechanical axis (or 9° to the anatomical axis) in the distal femoral resection to restore the natural mechanical axis and joint line. The rationale behind the AA is to enhance load distribution on the tibial component and improve patellar biomechanics by reducing the risk of lateral retinacular ligament stretching during knee flexion, along with reduced incidences of radiolucent lines on X-rays compared to the MA technique [36,37]. Technical limitations in the 1970s made it difficult to achieve precise bone cuts, potentially resulting in excessive (>3°) varus alignment of the limb or tibial implant, which hindered the widespread adoption of the AA. Due to concerns about accuracy and polyethylene wear, the AA has gradually declined in use over the past 40 years, with MA becoming the preferred approach. However, these challenges have been addressed by two advancements: precision tools for implant positioning (such as navigation systems and robotic technology) and TKA implants with a built-in 3° obliquity in the joint line. This allows surgeons to achieve the benefits of AA through standard MA cuts, known as the "AA-like" technique. Both AA and AA alignment-like methods have shown promising mid to long-term results. Figure 2 illustrates the AA, the MA, and the KA.

The Adjusted Mechanical Alignment

This technique is a modification of the traditional MA approach, designed to partially correct the natural frontal deformity (varus or valgus) up to a maximum of 3°. This method maintains mild to moderate deformities while reducing more severe ones. Adjustments in implant positioning are primarily made on the femoral side, as advocates of aMA seek to keep the tibial implant aligned with the mechanical axis [38]. In practical terms, this alignment aims to achieve a target zone for HKA angles from 177° to 183°. To balance the knee, aMA allows adjustments of lateral distal femoral angle (LDFA) to range from 87° to 90° in valgus knees, and from 90° to 93° in varus knees, while maintaining a medial proximal tibial angle (MPTA) of 90°. When still outside the HKA angle target zone of 177°-183°, a medial or lateral soft tissue release may be performed [38]. Several noncomparative longitudinal studies have reported excellent functional outcomes and long-term survivorship for aMA in total knee arthroplasties for both varus and valgus knee conditions [39].

Functional Alignment

This is a surgical technique aimed at restoring the natural orientation and angle of the joint, guided by the soft-tissue envelope. This approach seeks to achieve personalized limb alignment within a 0°-3° safe range of coronal alignment, enabling patient-specific knee movement with minimal soft-tissue adjustments [40]. The method aligns components perpendicular to the femoral and tibial axes. Tools such as computer navigation or robotic systems are used to evaluate resection thickness, flexion-extension gaps, and overall limb alignment. Following the removal of osteophytes, varus or valgus forces are applied to restore natural soft-tissue tension and correct coronal plane deformities [41]. Adjustments may include valgus modification of the distal femoral resection and varus modification of the tibial resection, maintaining joint line height to improve knee flexion, patella tracking, and mid-flexion stability. FA is still in its early stages, with limited data available on long-term outcomes. Furthermore, it requires specialized technology and expertise.

Restricted Kinematic Alignment

This alignment has been developed by Vendittoli et al., and the main principles are to assess limb, femoral, and tibial components' frontal alignments with navigated and computer-assisted means, and restrict their indication of full KA technique, as proposed by Howell et al. for patients with a small constitutional frontal limb deformity (≤3°) and a distal femoral or proximal tibial frontal joint line with less than 5° obliquity [42]. The designer called this alignment range a safe alignment zone. When patients have extreme deformities outside the safe zone, designers perform bone cut adjustments to bring the patient into their safe zone of alignment [42]. Vendittoli et al. have proposed five rKA principles.

 1. Safe zone alignment or HKA boundaries: rKA surgeons should aim to reproduce individual lower limb anatomy while keeping the arithmetic HKA angle within ±3° range.

 2. Joint line orientation: the designer has proposed to reproduce individual anatomy while keeping the LDFA and the MPTA within ±5°. This stems from the fact that joint line orientation varies among the population, with the mean MPTA being 2.9° varus and the mean LDFA being 2.7° valgus. Furthermore, a study found that 80% had an LDFA and MPTA below 5° [42].

 3. Preservation of soft tissue envelope around the knee and avoid releases unless the patient's knee falls outside of principles 1 and 2. All soft tissues surrounding the knee, including the capsule, muscles, and ligaments, play an important role in knee kinematics. Ligaments' tension and laxity vary according to sex (females tend to be more lax), interindividual variations, and at different ranges of movements (not isomeric structures) [42].

 4. Femoral biomechanics and kinematics are the driving force in knee OA pathology. It should be preserved if possible, and corrections and adjustments should be made on the tibial side if necessary. In this principle, rKA designers and KA designers are in agreement in thinking that the femur is the main bone in OA cases.

 5. Pivot point: To limit the amount of bone resection in rKA knees, surgeons will resurface the unworn side (replacing it with the exact amount of implant thickness) and cut the worn side. This will create different pivot points at the coronal plane based on which compartment is affected by the OA changes. For example, a medial pivot point would resurface the medial compartment and modify the resection thickness on the lateral side (vice versa for a lateral pivot).

A potential limitation of rKA is that substantial bone cuts, either tibial and/or femoral, may be required to achieve the target alignment. A study has shown that significant adjustments/cuts were needed in 17% of knees (822 out of 4,800 osteoarthritic knees) [19]. Second, although early and mid-term results are promising, long-term data are limited and awaited.

Inverse Kinematic Alignment

In the rKA knee, the designer bases their philosophy on femur-first and driving force principle, whereas in the inverse kinematic alignment (iKA), a "tibia-first" technique aims to restore prearthritic MPTA within a target zone from 84° to 92° and HKA angle within a target zone from 174° to 183° [43,44]. The native HKA angle is found by correcting the coronal limb alignment for cartilage wear and/or bony erosion, or by tensioning the extension gap. To balance the knee, the LDFA is adjusted within a target zone from 84° to 93°. When still outside the HKA angle target zone of 174° to 183°, a medial or lateral soft tissue release may be performed [44].

PSI and Individual CMI

**Figure 3:**
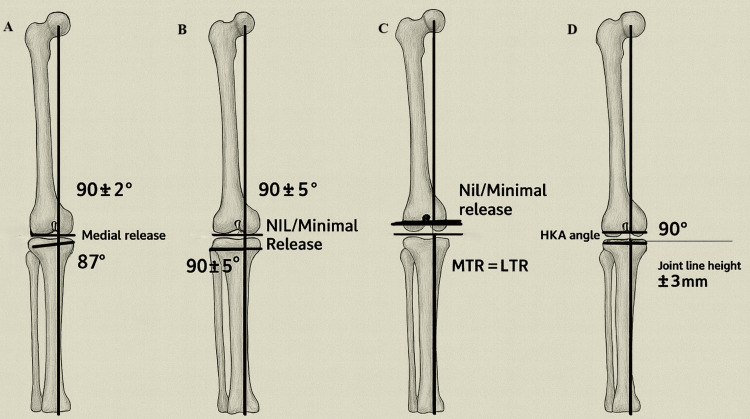
(A) Adjusted mechanical alignment, (B) restricted kinematic alignment, (C) inverse kinematic alignment, and (D) functional alignment MTR: medial tibial resection; LTR: lateral tibial resection; HKA: hip-knee-ankle Image credit: Created by the authors Mohammed Elmajee and Mahmoud Mersal using ChatGPT-4o image generation (OpenAI, www.openai.com) and edited in Adobe Illustrator (Adobe Inc.)

Patient-specific instrumentation (PSI) was introduced into knee arthroplasty around the beginning of this century, with custom-made implants (CMIs) following shortly thereafter, around 2006. There was no advantage over standard techniques regarding component alignment as well as clinical outcome 10-15 years following the introduction of the specific instrumentation (PSI) [45]. However, due to improved scanning and printing technology and utilization of MRI rather than CT, some promising early and mid-term results have been yielded [45]. CMIs have been compared with off-the-shelf implants in terms of pain, mobility, overall outcome and patient satisfaction, with studies showing no consistent superiority of CMIs over standard implants [46]. On balance, the literature shows mixed outcomes for alignment, implant positioning, and clinical outcomes so far; however, some advantages are commonly reported in the literature, including shorter operation times, reduced blood loss, and lower long-term costs [46-48]. Different techniques for aligning total knee arthroplasty implants are shown in Figure 3, which illustrates the aMA, the rKA, the iKA, and the FA. Except for the KA technique, all other techniques necessitate varying amounts of soft-tissue release (more so for systematic techniques than hybrid techniques) [1].

Kinematic alignment

KA is a newer philosophy that seeks to restore the patient's natural prearthritic anatomy by aligning the prosthetic components according to the individual's native joint lines and axes [34]. Rather than adhering strictly to the mechanical axis, KA focuses on reproducing the natural rotational and varus-valgus alignments of the femur and tibia, preserving the normal soft tissue tension and joint function [34]. KA aims to recreate the patient's natural knee movement by aligning the implant with the original orientation of the femur, tibia, and patella [34,49]. This approach seeks to minimize ligament releases, preserving the natural soft tissue balance and tension around the knee. The goal is to restore the patient's pre-disease knee alignment, which may not be a straight mechanical axis. There is limited long-term outcome data when compared to MA; however, early and mid-term results are promising [34].

KA TKR is resurfacing the arthritic knee or restoring the prearthritic knee kinematics. The more complicated definition is that KA co-aligns the axes of the joint lines of the component with the three kinematic axes and joint lines of the prearthritic or native knee without placing restrictions on the preoperative deformity, postoperative correction, and without ligament release [49].

**Figure 4:**
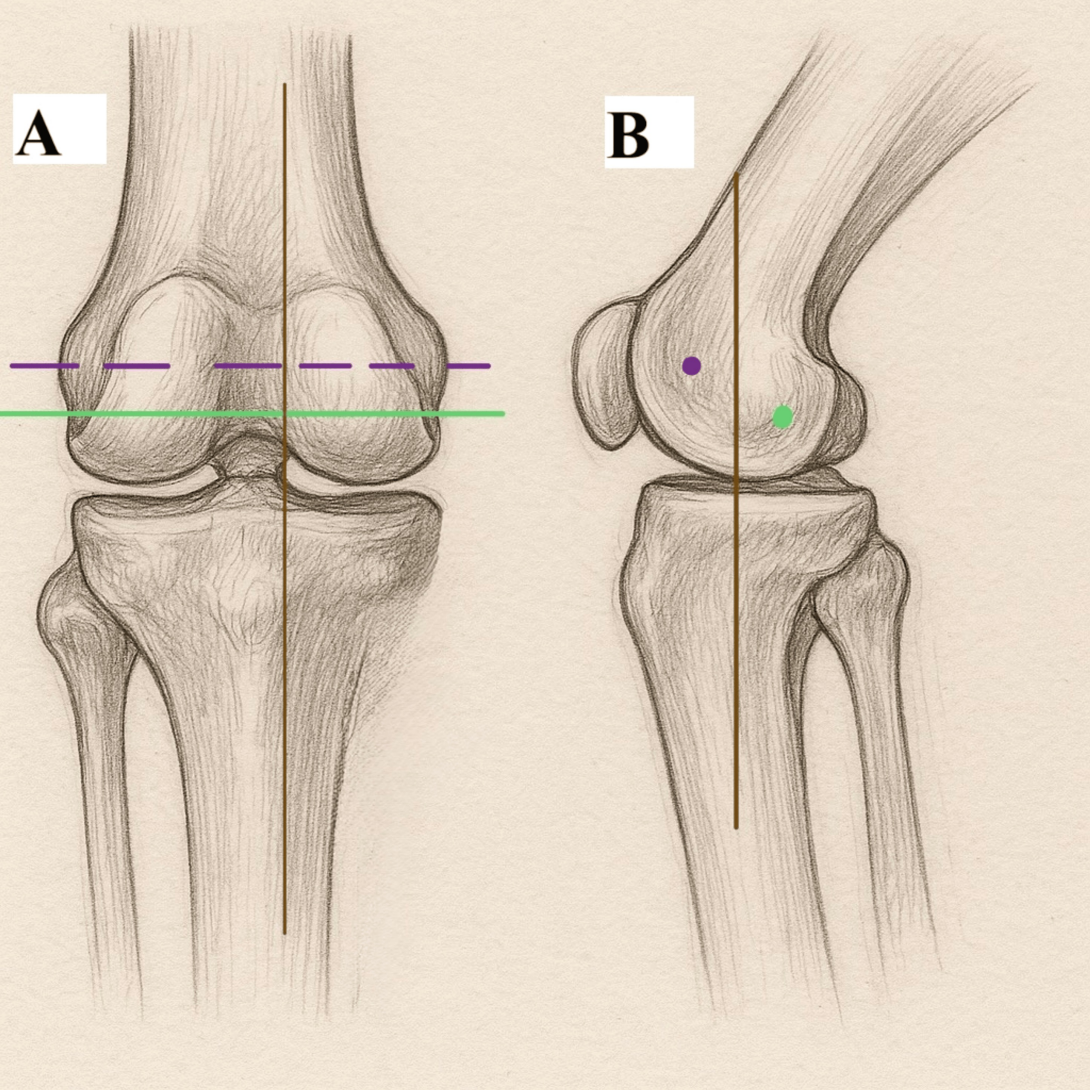
Different kinematic axes of the knee. (A) Posterior view showing the femoral flexion-extension axis (mauve dashed line), the tibial joint line axis (green line), and the longitudinal tibial rotation axis (brown vertical line). (B) Lateral view showing the femoral flexion-extension axis (mauve dot), the patellar flexion-extension axis (green dot), and the longitudinal tibial rotation axis (brown vertical line) Image credit: Created by the authors Mohammed Elmajee and Mahmoud Mersal using ChatGPT-4o image generation (OpenAI, www.openai.com) and edited in Adobe Illustrator (Adobe Inc.)

The three knee axes (two transverse axes and one vertical axis) on which the KA philosophy is based are the flexion-extension axis of the femur on the tibia, the patellar flexion and extension around the femur, and the internal and external rotation axis (the vertical axis) of the tibia on the femur [2,49,50]. The flexion-extension femoral tibial axis could be found by fitting two circles of equal radius on the medial and lateral femoral condyles, and the line connecting the centers of these circles is the flexion-extension femoral tibial axis, as shown in Figure 4. The brown line shows the longitudinal axis in the tibia that the tibia on the femur rotates about. In the femur, the green line shows the transverse axes about which the tibia flexes and extends. The mauve line indicates the transverse axis of the femur that the patella flexes and extends about.

The second transverse axis is the patellar flexion-extension axis. This axis is 10 mm proximal and 12 mm anterior and parallel to the other transverse axis [2]. The third axis is anterior and perpendicular to the other two transverse axes [3].

The resections performed in the KA knee should be equal to the thickness of the prosthesis implanted, after adjusting for the cartilage wear and kerf of the saw blade. In other words, ligaments and soft tissue envelope will not be changed; hence, the kinematics of the knee will be maintained. In performing KA knees, measuring the cuts made has more importance than adjusting intraoperative angles, as is the case with KA knees.

There are several issues related to all the alignment techniques mentioned above, and these issues have been discussed in the literature in some detail. One of these issues is the lack of robust, prospective, properly randomized, and well-controlled trials linking a knee alignment technique to PROM. Several factors have not been incorporated into the current studies and trials of knee alignment philosophies [51]. One of these issues is the accuracy and reproducibility of tools surgeons utilize to execute their planned alignment [52]. Prosthetic, long-term trials utilizing adjuncts such as robot-assisted surgery are warranted, with consideration given to the confounders such as obesity, previous surgery, extent of preoperative deformity, and the presence of neurological compromise. The second issue with what we already know about different alignment philosophies is that the majority of alignment research to date has concentrated on the coronal plane, yet true optimization requires viewing alignment as a triad encompassing coronal, sagittal, and axial planes. This comprehensive approach would help refine bone resection and prosthesis positioning.

Emerging techniques, such as adjusted mechanical, functional, and KA, reflect a shift toward individualized care. Although each method has its limitations, tailoring knee replacement to the native anatomy and preserving soft tissue shows promise.

## Conclusions

In conclusion, alignment philosophies in total knee arthroplasty have evolved from rigid mechanical techniques to more personalized approaches. While each method has benefits and limitations, the trend toward individualized alignment strategies shows promise in improving patient outcomes. Future high-quality studies are essential to define the most effective alignment techniques.
